# Transforming medical equipment management in digital public health: a decision-making model for medical equipment replacement

**DOI:** 10.3389/fmed.2023.1239795

**Published:** 2024-01-03

**Authors:** Luying Huang, Wenqian Lv, Qingming Huang, Haikang Zhang, Siyuan Jin, Tong Chen, Bing Shen

**Affiliations:** ^1^School of Health Science and Engineering, University of Shanghai for Science and Technology, Shanghai, China; ^2^Shanghai General Hospital, Shanghai, China; ^3^School of Medical Imaging, Shanghai University of Medicine & Health Sciences, Shanghai, China; ^4^Shanghai Tenth People’s Hospital of Tongji University, Shanghai, China

**Keywords:** medical equipment, decision-making, MCDM, game theory, hospital management

## Abstract

**Introduction:**

In the rapidly evolving field of digital public health, effective management of medical equipment is critical to maintaining high standards of healthcare service levels and operational efficiency. However, current decisions to replace large medical equipment are often based on subjective judgments rather than objective analyses and lack a standardized approach. This study proposes a multi-criteria decision-making model that aims to simplify and enhance the medical equipment replacement process.

**Methods:**

The researchers developed a multi-criteria decision-making model specifically for the replacement of medical equipment. The model establishes a system of indicators for prioritizing and evaluating the replacement of large medical equipment, utilizing game theory to assign appropriate weights, which uniquely combines the weights of the COWA and PCA method. In addition, which uses the GRA method in combination with the TOPSIS method for a more comprehensive decision-making model.

**Results:**

The study validates the model by using the MRI equipment of a tertiary hospital as an example. The results of the study show that the model is effective in prioritizing the most optimal updates to the equipment. Significantly, the model shown a higher level of differentiation compared to the GRA and TOPSIS methods alone.

**Discussion:**

The present study shows that the multi-criteria decision-making model presented provides a powerful and accurate tool for optimizing decisions related to the replacement of large medical equipment. By solving the key challenges in this area as well as giving a solid basis for decision making, the model makes significant progress toward the field of management of medical equipment.

## 1 Introduction

Large medical equipment is an essential material foundation for maintaining the normal operation of hospitals and improving their competitiveness (1, 2). With the iterative development of medical technology, hospitals should match the acquisition of medical equipment to the actual needs. In one survey, it was shown that nearly 60% of the total cost of a hospital project involves hospital equipment (3). The Malaysian government invested about MYR27 million in healthcare facilities in 2018 by implementing a program of new and upgraded medical equipment purchases (4). According to the Chinese government, the total value of medical equipment in all hospitals rose from RMB320 billion to RMB629 billion from 2010 to 2015, thus medical equipment occupies an important investment in public hospitals (5).

In practice, however, some hospitals blindly pursue the advancement of equipment, leading to unbalanced resource allocation and waste of resources. The phenomenon of under-utilization and over-utilization of equipment occurs repeatedly, adding an invisible burden to patients, reducing the operational efficiency of hospitals, and neglecting the actual needs of hospital work use (6, 7). Hospital management decision makers are faced with the challenge of replacing medical equipment in an orderly manner, especially when it comes to old equipment, and need to prioritise the replacement of various medical equipment through assessment and quantitative tools for effective allocation of state funds and the healthy development of clinical departments in hospitals (8). Too little or too slow replacement of equipment can easily lead to stagnation in the development of the department, hindering the healthy development of the hospital and affecting the patient’s experience.

Multiple Criteria Decision Making (MCDM) is a decision analysis method used to assist decision-makers in evaluating and selecting the best decision alternative among multiple decision criteria or standards (9). Evaluating major medical equipment replacement priorities is closely related to problem-solving using multicriteria decision-making (10). Due to the particular ambiguity and difficulty in defining indicators in solving multicriteria problems, MCDM calculates an overall score based on the weight of each criterion by quantifying the ranked quantitative criteria and provides effective decision-making on a more accurate basis (11). Common methods available include hierarchical analysis (AHP) (12), network analysis (ANP) (13), ideal solution similarity preference ranking (TOPSIS) (14), and data envelopment analysis (DEA). Presently, domestic and foreign scholars have thoroughly researched medical equipment replacement decisions. Mazloum Vajari S et al. (15) a proposed decision system that uses a hybrid SWOT-ANP-WASPAS approach provides solutions for medical equipment replacement programs. Ben Houria et al. (16) developed a multicriteria decision model based on AHP, TOPSIS and MILP methods to select the best maintenance strategy for the equipment by quantitatively ranking the different maintenance strategies of the equipment according to their importance. Mora-García T et al. (17) using an assessment tool based on multicriteria decision analysis, 12 indicators were defined for technical and economic aspects, resulting in the Medical Equipment Replacement Priority Indicator (MERUPI), which provides supporting criteria for deciding which medical equipment should be replaced and for the purchase plan. Faisal M et al. (18) proposed an analytical hierarchy processes -group decision-making (AHP-GDM) model, which includes 11 quantitative and qualitative indicators as primary and secondary criteria to prioritize medical equipment replacement priorities.

The focus of this paper is to develop a comprehensive MCDM model to test the feasibility and superiority of the improved COWA-PCA and GRA-TOPSIS methods in evaluating the replacement priorities of large medical equipment based on the example of four MRI devices.

## 2 Constructing the evaluation indicator system

This article follows the principles of systematicity, operability, independence and measurability (19), and combines the demand characteristics of the hospital and the technical characteristics of the equipment to construct an evaluation system for the replacement priority of large medical equipment, so as to assists hospitals and related departments in managing and replacing medical equipment more effectively (20, 21), ensuring that the equipment is operated efficiently, and to reduce the operating costs.

First, relevant data involving the renewal of large medical equipment were studied, and the indicators related to the equipment were initially screened. Subsequently, experts engaged in medical equipment management and hospital management were consulted using the Delphi method (22, 23), and the initially selected evaluation indicators were refined and perfected from the 20 indicator datasets based on the criteria of high sensitivity and collectability of the indicators. Finally, the evaluation index system was constructed from four aspects, namely, operation guarantee, social benefits, technical indicators (24) and economic benefits, in order to comprehensively evaluate the performance, efficiency and effectiveness of the equipment in actual operation, and to provide a more comprehensive and objective basis for the decision-making of equipment renewal, as shown in Table 1.

**Table 1:** Large medical equipment renewal priority evaluation indicator system.

First-level indicator	Second-level indicator	Meaning of indicator	Property
Operational security( $X_{1}$ )	Work Saturation( $X_{11}$ )	actual working load of the equipment	+
	Equipment Usage( $X_{12}$ )	actual working condition of the equipment	+
	Work Intensity( $X_{13}$ )	work density during the rated working time	+
Social benefits( $X_{2}$ )	Average Patient Waiting Time ( $X_{21}$ )	patient satisfaction with the examination	+
	Average Patient Interval Length ( $X_{22}$ )	efficiency of the equipment examination	+
Technical specifications( $X_{3}$ )	Life Index( $X_{31}$ )	current condition and performance of the equipment	+
	Failure Frequency( $X_{32}$ )	reliability of the equipment in actual operation	+
Economic benefits( $X_{4}$ )	Cost–benefit Ratio( $X_{41}$ )	ratio of revenue generated to operating costs	−
	Payback Period( $X_{42}$ )	payback time of the equipment investment	−

### 2.1 Operational security ( $X_{1}$ )

Operational assurance indicators reflect the stability and reliability of the equipment during actual use. These indicators allow us to understand how the equipment operates and whether it can meet the existing workload and demands.

1) Operating saturation ( $X_{11}$ ): higher operating saturation may lead To overworking of the equipment, thus affecting its stability and reliability.

(1) $$X_{11}=\frac{\left(\tau +2\right)\times \sum Z_{i}}{\eta_{b}}$$

Where τ refers to the average inspection time per patient, Z refers to the total number of inspections and $\eta_{b}$ the equipment’s powered-on runtime, and 2 refers to the preparation time set aside.

2) Equipment utilization ( $X_{12}$ ): a low equipment utilization rate may mean that equipment is sitting idle for a more extended period and resources are not being fully utilized.

(2) $$X_{12}=\frac{\eta_{w}}{\eta_{b}}$$

Where $\eta_{w}$ refers to the total working time of the equipment, from the start of the first patient’s inspection to the end of the last patient’s inspection.

3) Work intensity ( $X_{13}$ ): higher work intensities can lead to excessive wear and tear and equipment breakdowns

(3) $$X_{13}=\frac{\left(\tau +2\right)\times \sum Z_{i}}{\eta_{r}}$$

Where $\eta_{r}$ refers to the rated working time, generally 8 h daily.

### 2.2 Social benefits ( $X_{2}$ )

The social effectiveness indicators focus on patient satisfaction and quality of care. Understanding how well the equipment performs in meeting the needs of patients helps to evaluate the impact of the equipment on the overall reputation of the hospital and patient satisfaction. Societal benefits are important for decisions on equipment replacement.

1) Average patient waiting time ( $X_{21}$ ): longer waiting times may affect patient satisfaction and, thus, the impact of equipment on patient service quality.

(4) $$X_{21}=\frac{\sum \left(\tau_{\mathrm{bi}}-\tau_{ci}\right)}{\sum Z_{i}^{\prime }}$$

Where $\tau_{\mathrm{bi}}$ refers to the patient’s examination start time, $\tau_{ci}$ refers to the patient’s examination end time, $Z_{i}^{\prime }$ and refers to appointment times.

2) Average patient interval length ( $X_{22}$ ): longer examination intervals may mean that the equipment is insufficiently used, possibly due to poor appointment scheduling or operational delays resulting in longer waiting times for patients, which helps to understand the efficiency of the equipment.

(5) $$X_{22}=\sum \left(\tau_{\mathrm{bi}}-\tau_{e\left(i-1\right)}\right)$$

Where $\tau_{e\left(i-1\right)}$ refers to the previous patient’s end time.

### 2.3 Technical specifications ( $X_{3}$ )

Technical indicators focus on the technical performance and status of the equipment and can directly reflect the technical advancement and reliability. Technical indicators help to understand whether the equipment is at or near its expected service life and the failure rate of the equipment in actual operation.

1) Life index ( $X_{3}$ ): equipment has a reference useful life of 6 years, and a higher life index means that the equipment is close to or Has reached its estimated useful life, which can help in planning for replacement or upgrading of the equipment and affect the efficiency of the equipment

(6) $$X_{31}=\frac{\psi_{a}}{\psi_{p}}$$

Where $\psi_{a}$ refers to the current age of equipment, $\psi_{p}$ refers to the Estimated valid lifetimes of equipment, generally taken to 6 years.

2) Failure frequency ( $X_{32}$ ): a high number of failures may mean that the performance of the equipment decreases and helps to understand the stability and reliability of the equipment.

(7) $$X_{32}=\sum \phi_{b}$$

Where $\phi_{b}$ refers to several breakdown times.

### 2.4 Economic benefits ( $X_{4}$ )

The economic efficiency indicator looks at the equipment’s cost-effectiveness and investment recovery. It reflects the value of the equipment on an economic level and helps to determine whether the economic performance of the equipment is in line with expectations.

1) Cost–benefit ratio ( $X_{41}$ ): reflects the revenue generated To operating costs. A lower cost–benefit ratio may mean The equipment Is more expensive, reducing economic efficiency.

(8) $$X_{41}=\frac{R_{t}}{C_{t}}$$

Where $R_{t}$ refers to total equipment inspection revenue $C_{t}$ and equipment running costs, including staffing, maintenance, servicing, etc.

2) Payback period ( $X_{42}$ ): reflects the payback time of the equipment investment. A more extended payback period may mean a lower rate of return on the equipment and the need to delay the replacement of the equipment, helping to understand the economic value of the equipment and the benefits of the investment.

(9) $$X_{42}=\frac{C_{v}}{R_{t}-C_{t}}$$

Where $C_{v}$ refers to the price when the equipment was purchased.

## 3 Materials and methods

### 3.1 Research framework

**Figure 1:**
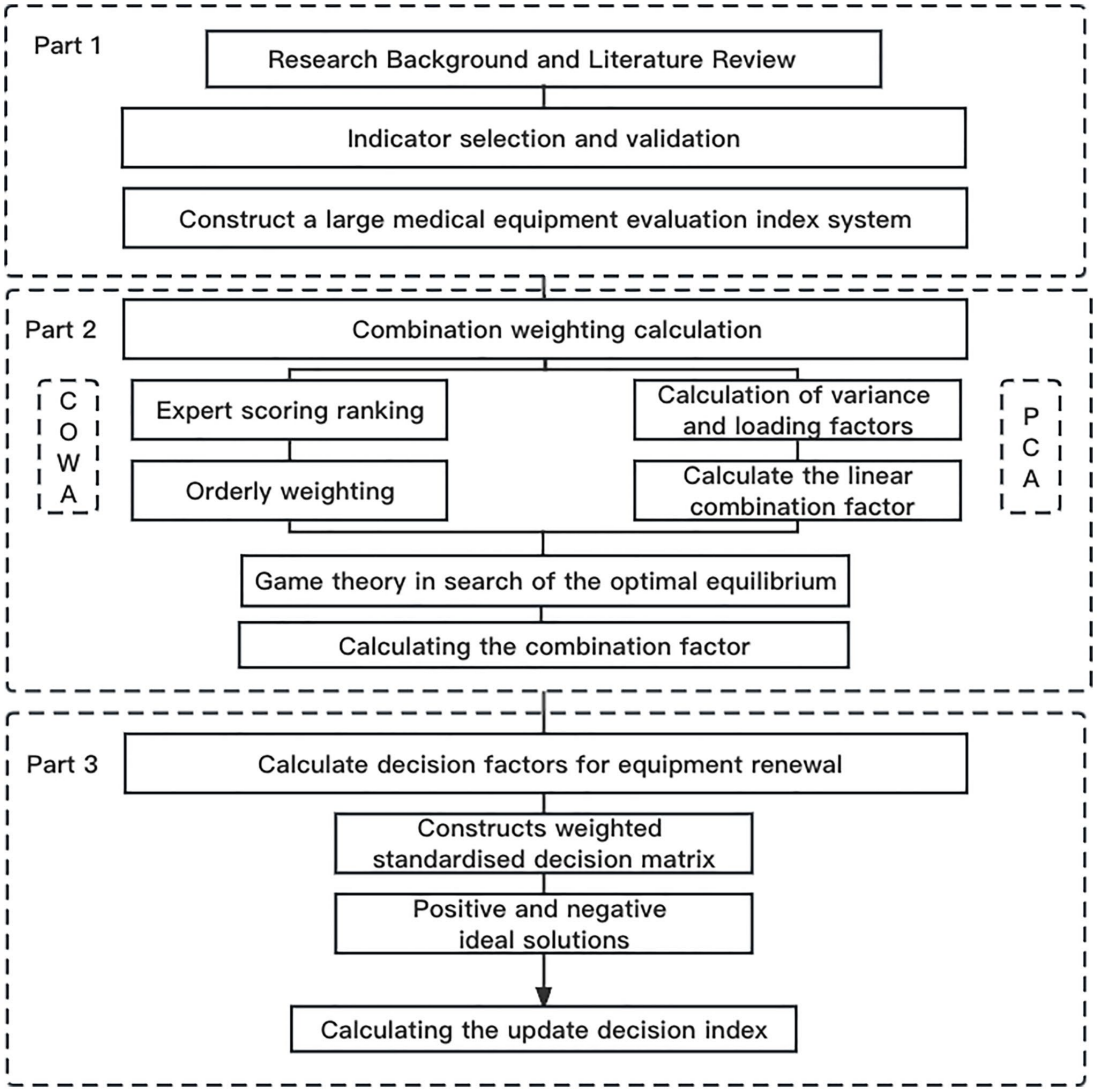
Flow chart.

The research framework of this article is shown in Figure 1. In Part 1, the evaluation indicator system for prioritizing the replacement of large medical equipment is constructed by looking up the literature as well as consulting with experts (see Table 2).

**Table 2:** Results of principal component analysis weights.

Indicator	Components 1	Component 2	Component 3	Component 4	Score	Weights
Characteristic roots	4.013	1.652	1.186	0.924		
Explanation of variance	44.59%	18.36%	13.18%	10.27%		
X_11_	0.443	0.158	0.297	0.218	0.333	0.124
X_12_	0.402	0.246	0.325	0.321	0.347	0.129
X_13_	0.392	0.195	0.123	0.335	0.302	0.113
X_21_	0.226	0.576	0.062	0.057	0.255	0.095
X_22_	0.358	0.151	0.229	0.348	0.293	0.109
X_31_	0.388	0.151	0.367	0.397	0.335	0.125
X_32_	0.097	0.612	0.336	0.046	0.237	0.088
X_41_	0.381	0.099	0.432	0.328	0.322	0.120
X_42_	0.065	0.339	0.549	0.588	0.259	0.096

In Part 2, COWA, PCA, and game theory methods (25) are combined to determine the weights of the indicators. The COWA method is mainly used to assign weights to different expert opinions to determine the key factors affecting the decision of equipment replacement. The PCA is used to calculate the objective weights of the indicators, and the raw indicators are transformed into principal components. The weights are determined based on the contribution of each principal component to the variance, thus reducing subjective bias. Game theory is used to promote balance and co-operation between multiple stakeholders to develop an optimal weight allocation strategy.

In Part 3, the GRA-TOPSIS method was used to prioritise equipment replacement. The relative correlation between equipment is determined by gray relational analysis (GRA) and the distance of each piece of equipment relative to the ideal solution is calculated using the TOPSIS method to prioritise the replacement decision. By integrating these methods, a combination of expert opinion, reducing information loss and analysing weighting relationships was achieved and the validity of the method was tested by comparing multiple methods and the Kendall method, ultimately providing excellent guidance for the decision-making process surrounding the replacement of large medical equipment.

### 3.2 Combined weighted averaging(COWA)

Text for this sub-section. C-OWA (26) is a method for determining experts’ weights. Based on the concept of Combined Ordered Weighted Average (OWA) and Compatibility Ranking (CO), the COWA operator first calculates the ranking compatibility of each expert on different evaluation indicators and then assigns weights to each expert based on the compatibility score. The method fully uses the experts’ experience, eliminates the negative effects of individual extremes, improves the scientific nature of the indicator assignment and avoids the extremes of the experts’ perceptions (27).

**Step 1:** by inviting n experts in the field of medical equipment to rate the importance of the indicators at each level (0 to 10 scale) , the initial scoring data of the experts from the data set $\left(a_{1},a_{2},\cdots \cdots ,a_{j},\cdots \cdots ,a_{n}\right)$ the scoring data in the data set are sorted from 0 to the smallest, and the result is $b_{0}\geq b_{1}\geq b_{2}\geq \cdots \geq b_{n-1}$ .

**Step 2:** using the combination number $b_{0}$ to determine the weights of the data, the weighting vector is applied to the decision data to obtain the absolute weights of the indicators $\bar{\omega_{i}}$ :

$\theta_{j+1}=\frac{C_{n-1}^{j}}{\sum_{k=0}^{n-1}C_{n-1}^{k}}=\frac{C_{n-1}^{j}}{2^{n-1}},j=0,1,2,\cdots ,n-1$ (10)

(11) $$\bar{\omega_{i}}=\overset{n-1}{\underset{j=0}{\sum }}\theta_{j+1}b_{j},i=1,2,\cdots ,m$$

Where $C_{n-1}^{j}$ is the combinatorial formula that calculates the number of methods to select j data from n−1 data, $2^{n-1}$ represents all possible combinations when selecting any quantity of data out of n−1 , $\theta_{j+1}$ offers a normalized weight for each j .

**Step 3:** Calculate the relative weights of the indicators $\omega_{i}$ , which achieved by normalizing the absolute weights $\bar{\omega_{i}}$ such that their aggregate is equal to 1.

(12) $$\omega_{i}=\frac{\bar{\omega_{i}}}{\sum_{i=1}^{m}w_{i}},i=1,2,\cdots ,m$$

Where $\overset{m}{\underset{i=1}{\sum }}w_{i}$ represents the summation of all absolute weights for all m indicators. This normalization ensures that the relative weights are proportional to the absolute weights, and their collective sum is 1, rendering them appropriate for scenarios where the relative significance between indicators is paramount.

### 3.3 Principal component analysis (PCA)

Principal Component Analysis (PCA) (28) is a method of multivariate statistical analysis in which multiple factors in an evaluation system are described by a few unrelated important variables, using linear equations to summarize and integrate all the factors so that they are used to reflect the variance at the higher level (29). All linear combinations are a type of principal component, and the information reflected in the selected composite factors is interpreted to make the overall evaluation model more balanced.

**Step 1:** The evaluation indicators are normalized to obtain a judgment matrix $\Upsilon_{ij}$

(13) $${ϒ}_{ij}=\frac{x_{ij}-x_{\text{min}}}{x_{\text{max}}-x_{\text{min}}},\left(i=1,\;2,\cdots ,n;\;j=1,\;2,\;\cdots ,m\right)$$

Where $x_{\text{max}}$ and $x_{\text{min}}$ are the highest and lowest values within a given unit.

**Step 2:** Calculate the correlation coefficient between the variables using the standardized data and calculate the correlation coefficient matrix $R={\left(r_{ij}\right)}_{m\times n}$

(14) $$r_{ij}=\frac{\sum_{k=1}^{n}y_{ki}y_{kj}}{n-1},\left(i=1,\;2,\cdots ,n;\;j=1,\;2,\cdots ,m\right)$$

**Step 3:** Calculate the eigenvalues and eigenvectors to solve for the eigenvalues and eigenvectors of the correlation coefficient matrix. The characteristic equation for the correlation moment $\left|R-\lambda E\right|=0$ calculates the eigenvalues $\lambda_{1},\lambda_{2}L,\lambda_{n}$ , and the non-zero solution $\left|R-\lambda E\right|X=0$ is the eigenvector $\mu_{1},\mu_{2}L,\mu_{n}$ .

**Step 4:** Calculate the variance contribution and cumulative variance contribution of each principal component:

(15) $$a_{j}=\frac{\lambda_{j}}{\sum_{j=1}^{n}\lambda_{j}},b_{p}=\frac{\sum_{k=1}^{p}\lambda_{k}}{\sum_{k=1}^{m}\lambda_{k}}$$

Where $a_{j}$ is the variance contribution, $b_{p}$ is the cumulative variance contribution， p is the number of principal components.

**Step 5:** Calculate the composite score:

(16) $$Z=\overset{p}{\underset{j=1}{\sum }}a_{j}P_{j}$$

Where P is the main component.

### 3.4 Game theoretical portfolio weights

In order to improve the objectivity, science and accuracy of the indicator assignment, game theory is introduced into the indicator weights (30). The game principle means that each player in the game decides which action to take, according to their interests and taking into account the possible impact of their decision-making behavior on the behavior of others, by considering the equilibrium between the mutually influencing behaviors, to achieve the goal of optimization of the subject’s objectives in a state of compromise between the factors (31). Therefore, game theory is introduced to consider the COWA and the PCA methods as two sides of the game, seeking the optimal combination of weights that will bring both sides to equilibrium. At this balance level, the sum of the deviations between the optimal combination weights and the two is minimized.

**Step 1:** Using *L* methods to determine the weights of the *n* indicators, the set of indicator weights is expressed as $W_{k}=\left(W_{k_{1}},W_{k_{2}},\cdots ,W_{k_{n}}\right)$ where k=1,2,⋯,L then the weight *L* vectors $W_{k}$ combination weights *w* are

(17) $$W=\overset{L}{\underset{k=1}{\sum }}\lambda_{k}W_{k},k=1,2,\cdots ,L$$

Where $\lambda_{k}$ is the linear combination factor.

**Step 2:** Minimize the divergence $\Delta =\left(W-W_{k}\right)$ between W and $W_{k}$ , and according to game theory principles, the corresponding optimization model is

(18) $$\min\overset{L}{\underset{k=1}{\sum }}{\left|\left|\lambda_{k}W_{k}^{T}-W_{k}\right|\right|}_{2},k=1,2,\cdots ,L$$

**Step 3:** Uses two weighting methods, taking the value L=2 . According to the principle of differentiation, the set of linear equations for the optimal first-order derivative condition is obtained by substituting Eq:

(19) $$\left[\begin{array}{cc} W_{1}W_{1}^{T} & W_{1}W_{2}^{T}\\ W_{2}W_{1}^{T} & W_{2}W_{2}^{T} \end{array}\right]\left[\begin{array}{c} \lambda_{1}\\ \lambda_{2} \end{array}\right]=\left[\begin{array}{c} W_{1}W_{1}^{T}\\ W_{1}W_{2}^{T} \end{array}\right]$$

**Step 4:** The combination coefficients $\lambda_{1}$ and $\lambda_{2}$ are obtained according to Eq. and normalized to $\lambda_{k}^{*}$ , which in turn gives the game combination weights $W^{*}$ :

(20) $$W^{*}=\lambda_{1}W_{1}^{T}+\lambda_{2}W_{2}^{T}$$

### 3.5 Gray relational analysis and technique for order of preference by similarity to ideal solution (GRA-TOPSIS)

Text for this sub-section, the TOPSIS method (32) is suitable for applying large multi-factor systems, which avoids the subjectivity of data and describes the overall evaluation of multiple factors. GRA (33) can be used to judge an indicator’s merits by the degree of similarity in geometric shape trends between factors. Each of the above two methods has its advantages (34). The combination of the weighted TOPSIS and GRA to construct a medical equipment replacement priority model makes the conclusions of the model calculation more consistent with practice and scientific.

**Step 1:** Construct a weighted normalized decision matrix S , calculate the Euclidean distance to the positive and negative ideal solutions for each evaluation object $d_{i}^{+},d_{i}^{-}$ :

(21) $$S={\left(s_{ij}\right)}_{m\times n}={\left(w_{j}\times z_{ij}\right)}_{m\times n^{\prime }}$$

(22) $$\begin{array}{l} d_{i}^{+}=\sqrt{\overset{n}{\underset{j=1}{\sum }}{\left(s_{ij}-s_{j}^{+}\right)}^{2}},\\ d_{i}^{-}=\sqrt{\overset{n}{\underset{j=1}{\sum }}{\left(s_{ij}-s_{j}^{-}\right)}^{2},}i=1,2,\cdots ,m,j=1,2,\cdots ,n \end{array}$$

$$\mathrm{Where}s_{j}^{+}=\max_{j}s_{ij};s_{j}^{-}=\min_{j}s_{ij}\text{.}$$

**Step 2:** Calculate the matrix of gray correlation coefficients between each solution and the positive and negative ideal solutions:

(23) $$H^{+}={\left\{h_{ij}^{+}\right\}}_{m\times n^{\prime }},H^{-}={\left\{h_{ij}^{-}\right\}}_{m\times n^{\prime }}$$

(24) $$h_{ij}^{+}=\frac{{\mathrm{minmin}}_{i}\left|s_{j}^{+}-s_{ij}\right|+]\rho {\mathrm{maxmax}}_{i}\left|s_{j}^{+}-s_{ij}\right|}{\left|s_{j}^{+}-s_{ij}\right|+\rho \max_{i}\max_{j}\left|s_{j}^{+}-s_{ij}\right|}$$

(25) $$h_{ij}^{-}=\frac{{\mathrm{minmin}}_{i}\left|s_{j}^{-}-s_{ij}\right|+{\mathrm{maxmax}}_{i}\left|s_{j}^{-}-s_{ij}\right|}{\left|s_{j}^{-}-s_{ij}\right|+\rho \max_{i}\max_{j}\left|s_{j}^{-}-s_{ij}\right|}$$

Where ρ is the resolution factor and $\rho \in \left(0,1\right)$ is taken from ρ=0.5 .

**Step 3:** Calculate the gray correlation coefficients of each evaluation object and the positive and negative ideal solutions $l_{i}^{+},l_{i}^{-}$ , and dimensionless process the Euclidean distance $d_{i}^{+},d_{i}^{-}$ and the correlation $l_{i}^{+},l_{i}^{-}$ :

(26) $$l_{i}^{+}=\frac{1}{n}\overset{n}{\underset{j=1}{\sum }}W_{j}^{*}h_{ij}^{+},l_{i}^{-}=\frac{1}{n}\overset{n}{\underset{j=1}{\sum }}W_{j}^{*}h_{ij}^{-}$$

(27) $$D_{i}^{+}=\frac{d_{i}^{+}}{\underset{i}{\max^{+}}},D_{i}^{-}=\frac{d_{i}^{-}}{\underset{i}{\bar{\max}}}$$

(28) $$L_{i}^{+}=\frac{l_{i}^{+}}{\underset{i}{\max^{+}}},L_{i}^{-}=\frac{l_{i}^{-}}{\underset{i}{\bar{\max}}},i=1,2,3,\cdots m$$

**Step 4:** Combine the Euclidean distance $D_{i}^{+},D_{i}^{-}$ and the gray correlation coefficient $L_{i}^{+},L_{i}^{-}$ and calculate the replacement decision factor $\xi_{i}$ :

(29) $$T_{i}^{+}=\alpha_{1}D_{i}^{-}+\alpha_{2}L_{i}^{+},T_{i}^{-}=\alpha_{1}D_{i}^{+}+\alpha_{2}L_{i}^{-}$$

(30) $$\xi_{i}=\frac{T_{i}^{+}}{T_{i}^{+}+T_{i}^{-}},i=1,2,3,\cdots m$$

Where $\alpha_{1},\alpha_{2}$ reflects the decision maker’s preference for location and shape, and $\alpha_{1}+\alpha_{2}=1,\alpha_{1},\alpha_{2}\in \left[0,1\right]$ . $\alpha_{1},\alpha_{2}$ values are empirically taken as 0.5 in general. The more significant the corresponding replacement decision factor $\xi_{i}$ , the better the object; the smaller the corresponding replacement decision factor $\xi_{i}$ , the worse the object.

## 4 Results

### 4.1 Case presentation and data sources

Text for this sub-section. Four magnetic resonance imaging (MRI) machines in the hospital are used alternately with old and new equipment. The equipment types are Signa HDx, MR750, MR750W and Prisma, located in different parts of the hospital.

In order to fully access the evaluation indicators for equipment replacement, multiple data sources are used to ensure the accuracy and completeness of the required information. Firstly, data collectors were installed on the large equipment to collect critical data such as hours of operation, start-up time and workload in real-time. These data collectors use advanced image recognition technology to accurately monitor the on/off status of the equipment. At the same time, the data collectors read information from the equipment’s examination interface through a frequency divider and use image recognition technology to identify essential information such as patient numbers and examination sequences, enabling accurate calculation of the equipment’s total working examination time.

In order to obtain more effective data, the real IoT collection data is matched with other information systems in the hospital as well as data integration. By interfacing with PACS, HIS, RIS, reservation system, ERP and other related systems, data acquisition of data fields required by indicators is achieved, to obtain metric fields such as patient appointment days, average interval length, and revenue. Through in-depth analysis of patient payments and examination moments in these systems, key indicators such as patient appointment days, average interval length, and revenue were obtained. This process uses rigorous data cleaning and validation methods to ensure the reliability and accuracy of the data. In addition, a close working relationship is maintained with the equipment manufacturers to obtain data on failures data. Equipment manufacturers regularly provide information on equipment failure fills, which is carefully verified and collated to provide a reliable data source for assessing equipment failure count indicators.

In summary, the multiple data sources and rigorous data processing methods ensure that the indicator data used in assessing equipment replacement decisions are accurate and comprehensive. This provides a solid study database and helps make more scientific and rational equipment replacement decisions.

### 4.2 Correlation analysis

**Figure 2:**
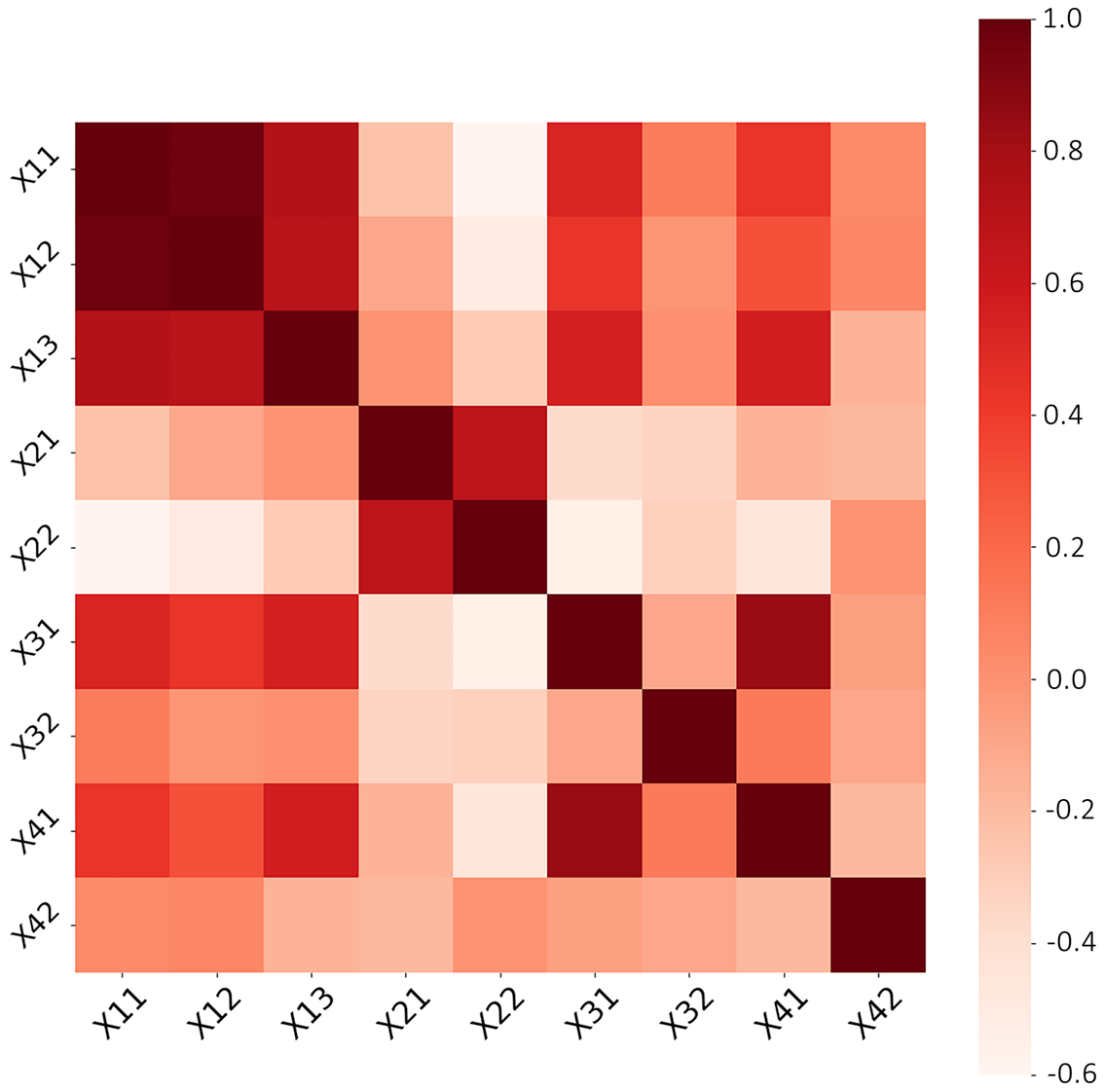
Indicator correlation.

Text for this sub-section. Pearsons’ method in SPSS is used to calculate correlations between indicators. It can reveal the strength and direction of linear relationships between variables, which indicators have strong positive or negative correlations with each other (35), to understand the interactions between indicators and provide a basis for the subsequent principal component analysis. The result is shown in Figure 2.

The evaluation indicators were subjected to Pearson correlation analysis to reveal their interrelationships. The analysis results show a high positive correlation between equipment utilization, work saturation and work intensity, indicating that these indicators may influence each other. The payback period was positively correlated with the average interval length, indicating that the average interval length also tends to increase when the payback period increases. In contrast, there is a negative correlation with equipment utilization, work saturation, average appointment length, work intensity, equipment life index and cost–benefit ratio, indicating that these indicators tend to decrease when the payback period increases. The average appointment length positively correlated with the number of breakdowns, suggesting that more equipment breakdowns may lead to increased appointment length.

### 4.3 Determination of weights

**Figure 3:**
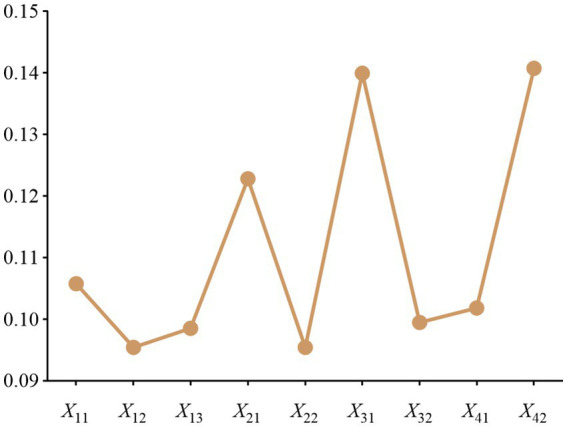
Experts Scoring.

Text for this sub-section. To assess the importance of the indicators, eight experts in the field of medical device management were invited to score the indicators and weight them using the C-OWA method. The scores are shown in Figure 3.

**Figure 4:**
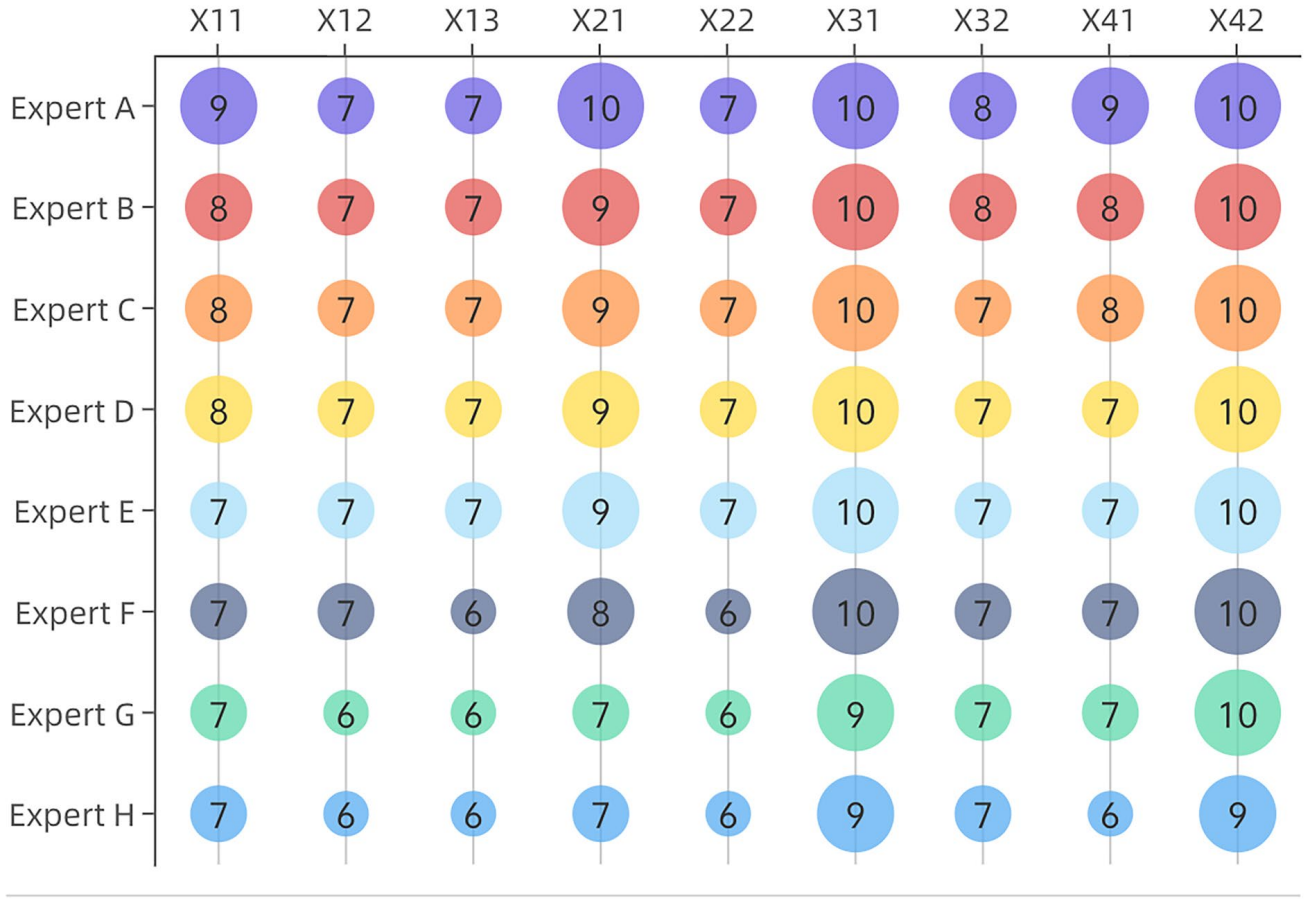
COWA weighting results.

Expert scores (0–10) for the evaluation indicators covered in this paper, with larger scores representing higher importance of the indicators. Take indicator X_11_ as an example, sort the scores of indicator X_11_ from the largest to the smallest to get (7–9), and calculate the weight vector by the formula to get: (0.07, 0.44, 1.31, 2.19, 2.19, 1.15, 0.38, 0.05). The results of the weight values are shown in Figure 4.

**Figure 5:**
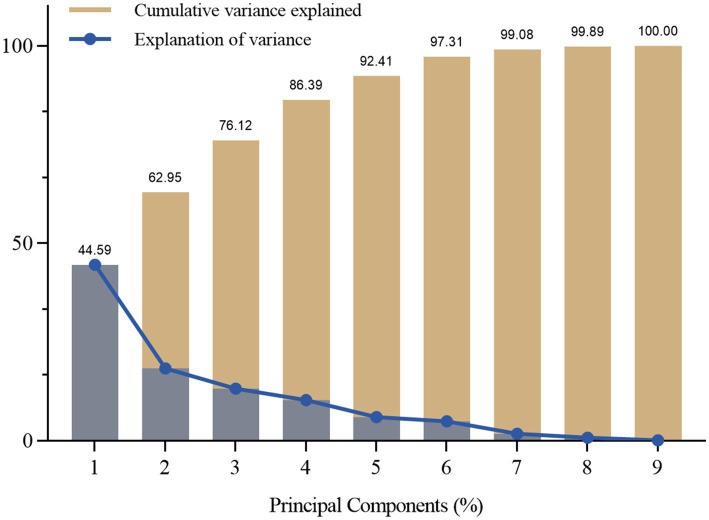
Principal component feature results.

As shown in Figure 5, the gravimetric plot is a visual tool commonly used to present the results of principal component analysis, determining the number of principal components that should be retained. The graph shows the percentage of variance explained by each principal component and the cumulative percentage of variance explained. An inflection point can be found where the number of principal components retained explains most of the variance in the original data while avoiding the problem of overfitting due to retaining too many principal components. The curve begins to level off at the fourth eigenvalue point. The variance explained by these four principal components are 44.59, 18.36, 13.18 and 10.27% respectively, and the cumulative variance explained is 86.39%.

**Figure 6:**
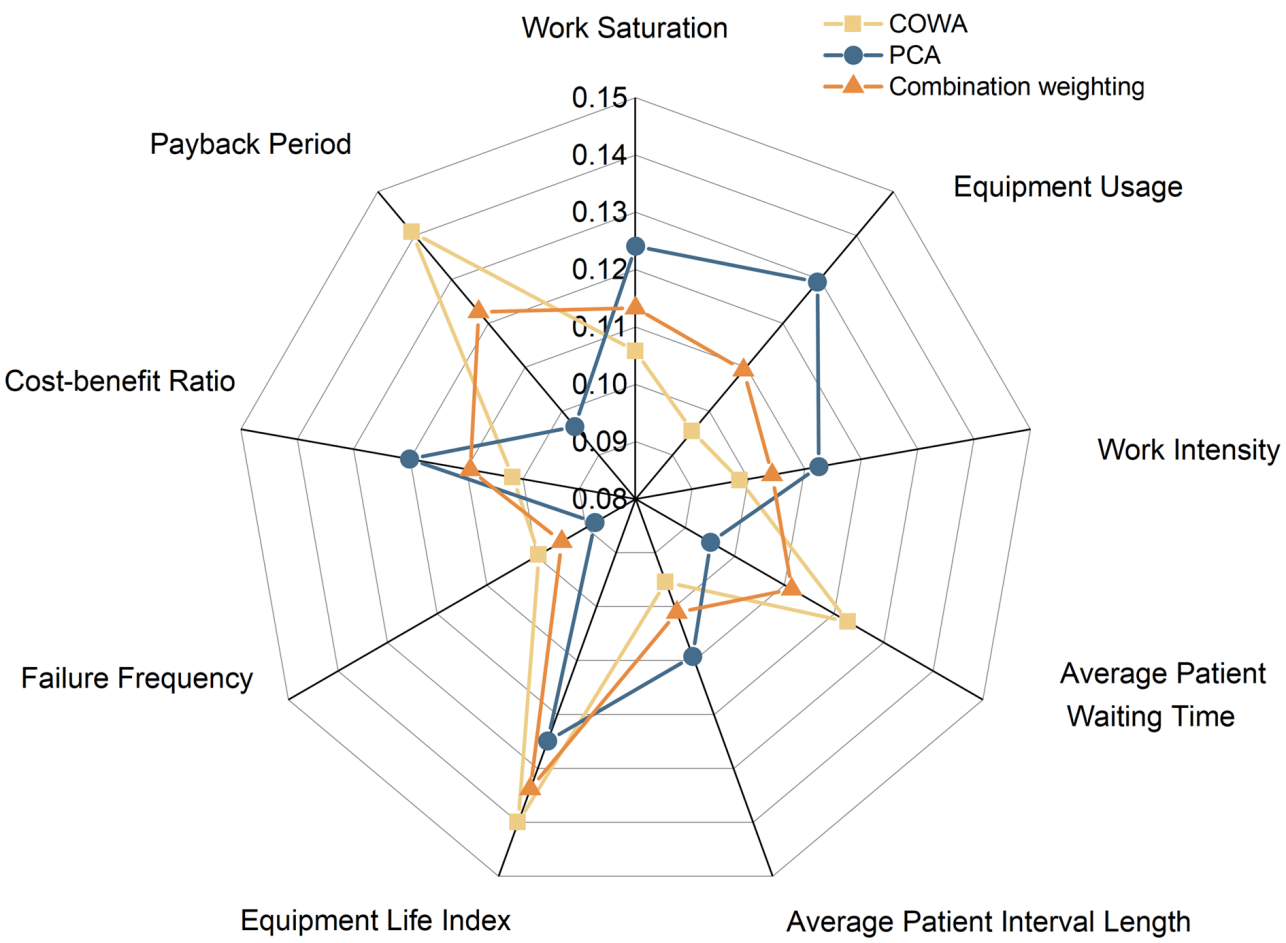
Comparison of weight values for different methods.

After calculating the PCA weights and COWA subjective weights, these weights were combined using a game-theoretic approach to obtain a combined weight value for each evaluation indicator. The comparison of the weights calculated by the three methods is shown in Figure 6, which takes into account both the variability of the evaluation indicators and the conflicting and variable nature of the indicator data, reflecting the objectivity of the data itself and helping to provide more targeted guidance to decision-makers, making the weight calculation results more reasonable.

### 4.4 Updating decision factor determination

Text for this sub-section. The Euclidean distances of the positive and negative ideal solutions $s_{j}^{+},s_{j}^{-}$ and the positive and negative ideal solutions $d_{i}^{+},d_{i}^{-}$ can be obtained by using Eq. The gray correlation matrix $H^{+}={\left\{h_{ij}^{+}\right\}}_{m\times n^{\prime }},H^{-}={\left\{h_{ij}^{-}\right\}}_{m\times n^{\prime }}$ and the correlation degree $l_{i}^{+},l_{i}^{-}$ are calculated for each device according to Eq. The correlations between the indicators are reflected in the three-dimensional space. Furthermore, the Euclidean distances $D_{i}^{+},D_{i}^{-}$ and the correlations $L_{i}^{+},L_{i}^{-}$ for each device are processed dimensionless according to (26)–(28). The results are shown in Table 3.

**Table 3:** Results of principal component analysis weights.

Equipment Type	European distance		Gray correlation	
	Positive	Negative	Positive	Negative
Signa HDx	1.000	0.646	0.873	1.000
MR750	0.622	0.985	0.979	0.875
MR750W	0.410	1.000	1.000	0.818
Prisma	0.570	0.959	0.982	0.859

**Figure 7:**
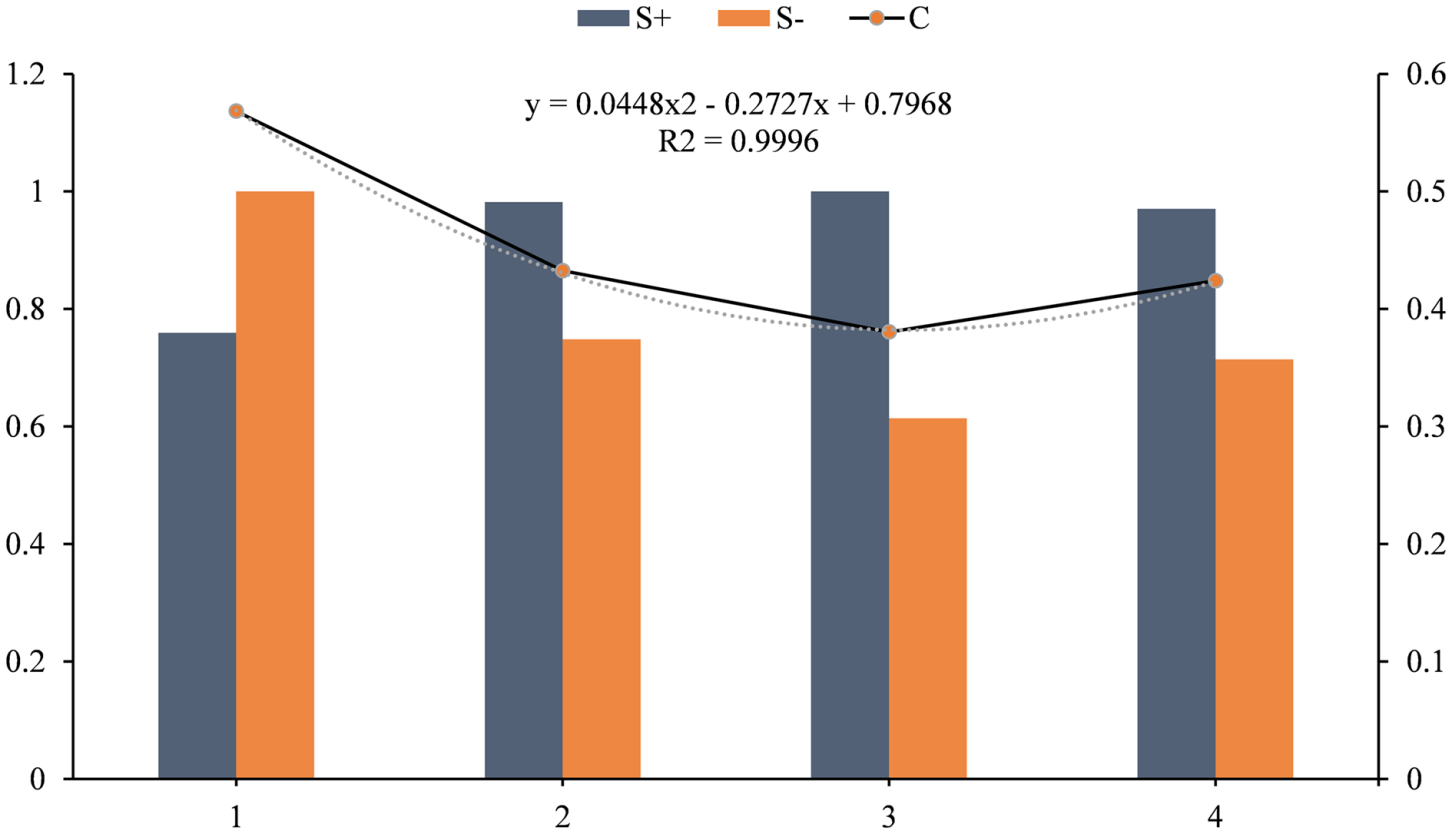
Updating decision results.

The more significant the replacement decision factor, the more the device needs replacement. The results of ranking the four MRI devices using the combined weight GRA-TOPSIS evaluation model are SignaHDx < MR750 < Prisma < MR750W. The GRA-TOPSIS model calculates a uniform and reasonable distribution of the resulting values, shown in Figure 7.

The VIKOR method (36) is also applicable to multi-attribute decision problems and is able to take into account the complementarity and conflict between attributes when determining the weights of each attribute. This article compares the results of the calculations of several methods of the GRA method, TOPSIS method, GRA-TOPSIS method and VIKOR method were used to calculate the replacement priority of large medical equipment, respectively, and the data were standardized to take complete account of the closeness of individual indicators to the indicator series. The replacement decision results and ranking results are shown in Table 4.

**Table 4:** Updating decision results.

Equipment type	GRA		TOPSIS		GRA-TOPSIS		VIKOR	
	Score	Sort	Score	Sort	Score	Sort	Score	Sort
Signa HDx	0.864	1	0.613	1	0.568	1	0.000	1
MR750	0.756	3	0.393	2	0.433	2	0.831	3
MR750W	0.707	4	0.296	4	0.380	4	0.807	2
Prisma	0.742	2	0.379	3	0.424	3	0.990	4

**Figure 8:**
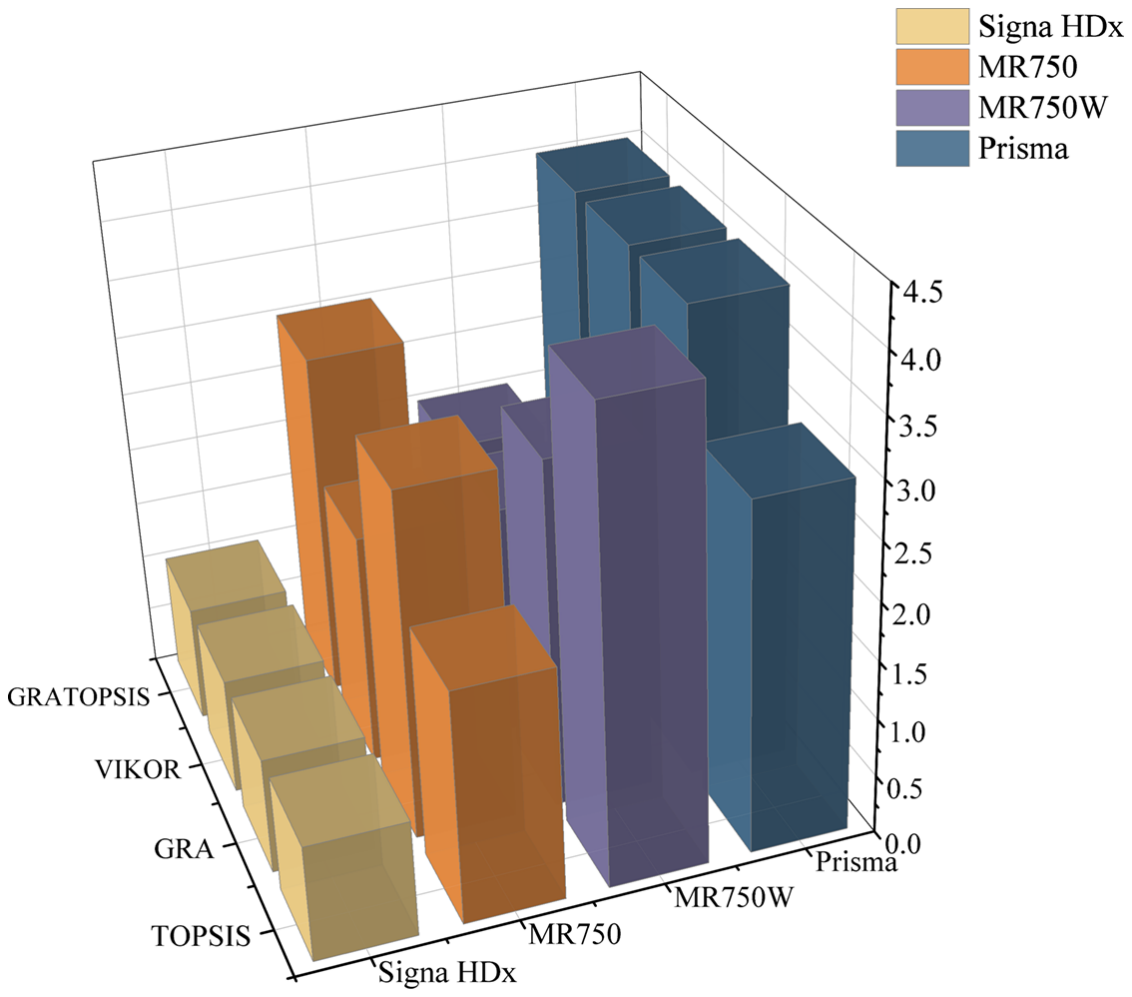
Sorting chart of replacement results.

The results in the table show that compared to the GRA and TOPSIS methods alone, the GRA-TOPSIS method has significantly improved in terms of correlation coefficients, thus better coping with the uncertainties and limitations in the evaluation process. The accuracy of the traditional GRA method may be limited when dealing with decision problems with complex quantitative attributes. In contrast, the accuracy of the TOPSIS method suffers when correlations exist between attributes. The VIKOR method is not applicable when the result values differ too much in the replacement decision evaluation process. This suggests that the GRA-TOPSIS method has higher accuracy and reliability in evaluating replacement priorities for large medical equipment. A comparison of the results obtained from the four methods is shown in Figure 8.

Kendall’s test (37) can measure the correlation between multiple evaluation methods and assess their consistency in solving the problem of prioritizing the replacement of large medical equipment, which helps to reveal the similarities and differences between different methods in terms of evaluation results so that the best evaluation method suitable for the actual problem can be selected in a targeted manner. Kendall’s test was used to analyze the consistency of the three methods, GRA, TOPSIS and GRA-TOPSIS, to better understand the strengths and weaknesses of the different methods and to provide a reference for subsequent research, which is shown in Table 5.

**Table 5:** Kendall’s result.

Methods	Rank average	Median	Kendall’s W	*X* ^2^	*p*
GRA	3	0.749	0.812	6.5	0.039
TOPSIS	1.25	0.386			
GRA-TOPSIS	1.75	0.428			

The table gives Kendall’s W coefficient of 0.812, an X^2^ value of 6.5 and a value of p of 0.039. Kendall’s W coefficient is close to 1, indicating a high level of consistency in the replacement decision factors between the three methods. Also, as the value of p is less than 0.05, the overall data level shows significance. Therefore there is a significant correlation between the three methods regarding the replacement decision factor.

## 5 Discussion

This research constructs a large medical equipment renewal priority evaluation indicator system, covering four aspects: operational security, social benefits, technical indicators and economic benefits. This system is designed to provide a robust framework for medical equipment administrators and policymakers, thereby facilitating empirically informed renewal decisions. The employed methodology harnesses the combined strengths of the COWA and PCA methods, bridging both subjective judgment and objective data attributes. Through an amalgamation of subjective weights derived from COWA with objective weights from PCA, realized via game-theoretic reasoning, a comprehensive weighting system is established. This approach not only minimizes the potential for information loss, which is often inherent in isolated weighting schemes, but also augments error resilience and alignment, ensuring enhanced methodological precision and relevance. Building on this foundational methodology, the research introduces an integrative multicriteria decision model, combining the attributes of COWA-PCA and GRA-TOPSIS. The GRA method, adept at handling sparse and fragmented data sets, aligns seamlessly with the attributes of TOPSIS, which excels in analyzing multi-attribute decision matrices. The union of gray correlation (from GRA) with Euclidean distance (from TOPSIS) ensures a model capable of addressing both uncertainty and information incompleteness. This fusion not only circumvents the limitations of unilateral approaches but also amplifies the accuracy and robustness of evaluations regarding equipment renewal priorities.

Significant preparation and groundwork has been invested to ensure that the research has solid utility and enhances the full lifecycle management of large medical devices. The deployment of advanced IoT collectors, the development of interfaces with third-party systems, and rigorous database management have combined to create a robust dataset. Although the proposed model requires some computational power and computational cost, in terms of economic and social benefits, the model can better guide the management of medical devices, thus saving costs and improving service quality for healthcare organizations, and bringing considerable economic dividends to hospitals, thus achieving greater economic benefits. The scalability and replicability of this project offer great prospects for subsequent data-centric exploration in this area.

For empirical validation, data from MRI equipment in a selected third-tier hospital was utilized. The results revealed that the GRA-TOPSIS method returned an R^2 value of 0.9667, attesting to its alignment with empirical realities. In addition, Kendall’s test validates the robustness of GRA-TOPSIS with respect to the GRA and TOPSIS methods, emphasizing its usefulness as an effective tool for decision-making on equipment replacement in various industries.

## 6 Conclusion

This article constructs a comprehensive medical equipment replacement priority evaluation indicator system and proposes a comprehensive multicriteria decision model based on COWA-PCA and GRA-TOPSIS. In practical application, the medical equipment replacement priority evaluation model proposed in this paper can help hospital managers and policymakers to better understand and evaluate the need for equipment replacement so as to formulate more scientific and rational replacement strategies. Comparing and analyzing the equipment replacement priorities of different hospitals can provide a basis for resource allocation and policy formulation. In addition, the findings of this paper can also inspire equipment replacement decisions in other fields.

In future, research could further expand the study area and methodology to include a wider and more diverse range of data sources, diversifying the range of evaluation metrics to capture a broader range of operational realities and patient-centered outcomes. Continuous refinement and integration of evaluation methods is essential to improve the accuracy, robustness and generalisability of evaluation models. In addition, with the development of big data technology, artificial intelligence technology and other technologies (38, 39), in hopes of building an intelligent management platform for large medical equipments, ensuring a more scientific, rational and effective decision-making process driven by data. Through in-depth research and practical application, it is expected to provide more scientific, rational and effective support for medical equipment decision-making.

## Data availability statement

The raw data supporting the conclusions of this article will be made available by the authors, without undue reservation.

## Author contributions

LH, WL, and BS: Conceptualization. LH, QH, and HZ: methodology. LH, WL, and QH: validation. LH, WL, and HZ: formal analysis. QH and SJ: investigation. BS, TC, and WL: resources. TC and WL: data curation. LH and WL: writing—original draft preparation. BS, HZ, and QH: writing—review and editing. LH and SJ: visualization. BS: supervision. All authors contributed to the article and approved the submitted version.
